# Epidemiological Features and Forecast Model Analysis for the Morbidity of Influenza in Ningbo, China, 2006–2014

**DOI:** 10.3390/ijerph14060559

**Published:** 2017-05-25

**Authors:** Chunli Wang, Yongdong Li, Wei Feng, Kui Liu, Shu Zhang, Fengjiao Hu, Suli Jiao, Xuying Lao, Hongxia Ni, Guozhang Xu

**Affiliations:** 1 Department of Chronic Diseases and Community Health, Fenghua Municipal Center for Disease Control and Prevention, Ningbo 315500, China; chunliwangx@163.com (C.W.); fhfengw@163.com (W.F.); 2 Department of Virus Research, Ningbo Municipal Center for Disease Control and Prevention, Ningbo 315010, China; nbliyd@163.com (Y.L.); nbzhangs@163.com (S.Z.); nbhufj@163.com (F.H.); nbjiaosl@163.com (S.J.); nblaoxy@163.com (X.L.); nbnihx@163.com (H.N.); 3 Department of Science Research and Information Management, Zhejiang Provincial Center for Disease Control and Prevention, Hangzhou 310051, China; zjliuk@163.com

**Keywords:** ARIMA model, influenza, influenza-like illness, prediction

## Abstract

This study aimed to identify circulating influenza virus strains and vulnerable population groups and investigate the distribution and seasonality of influenza viruses in Ningbo, China. Then, an autoregressive integrated moving average (ARIMA) model for prediction was established. Influenza surveillance data for 2006–2014 were obtained for cases of influenza-like illness (ILI) (*n* = 129,528) from the municipal Centers for Disease Control and virus surveillance systems of Ningbo, China. The ARIMA model was proposed to predict the expected morbidity cases from January 2015 to December 2015. Of the 13,294 specimens, influenza virus was detected in 1148 (8.64%) samples, including 951 (82.84%) influenza type A and 197 (17.16%) influenza type B viruses; the influenza virus isolation rate was strongly correlated with the rate of ILI during the overall study period (*r* = 0.20, *p* < 0.05). The ARIMA (1, 1, 1) (1, 1, 0)_12_ model could be used to predict the ILI incidence in Ningbo. The seasonal pattern of influenza activity in Ningbo tended to peak during the rainy season and winter. Given those results, the model we established could effectively predict the trend of influenza-related morbidity, providing a methodological basis for future influenza monitoring and control strategies in the study area.

## 1. Introduction

Influenza is an acute, strongly infective respiratory disease caused by the influenza virus [1]. The epidemic and pandemic forms are major health threats to humans. Disease surveillance, including monitoring of influenza-like illness (ILI) and influenza virus infections, plays a significant role in controlling and preventing influenza epidemics and pandemics [2].

In many parts of the world, particularly in developed regions, the etiologic agents associated with ILI have been well characterized [3]. However, the epidemiology and etiology of ILI are poorly understood in developing countries, creating challenges for governments when planning interventions and prevention strategies. The lack of data also inhibits the modeling of pandemic influenza infections and the development of appropriate control strategies.

Ningbo is located in the middle of the coastline of Mainland China, adjacent to the East China Sea, and has a subtropical climate with some seasonal variability in temperature. The hottest period (rainy season) extends from June to September, and the coldest period occurs from November to February. Although seasonal influenza occurs annually in Ningbo, there have been no studies regarding the prediction of the associated monthly morbidity. Early warning based on forecasts is very important for improving disease control, community intervention, and personal protection.

Increasing studies have applied model methods to identify the potential mechanics of communicable diseases, such as the linear regression method [4], transmission model method [5], and gray model method [6]. Of the popular methods currently used for the prediction of influenza, the autoregressive integrated moving average (ARIMA) model is one of the most widely used time series models [7]. The ARIMA model has several advantages for forecasting compared with other methods, such as a moving average, exponential smoothing, and a neural network. It is very useful in modeling the temporal dependence structure of a time series [8,9].

In this study, the epidemiological and etiology characteristics of patients with ILI symptoms during 2006–2014 in Ningbo, China were studied to identify circulating influenza virus strains and vulnerable population groups and to investigate the distribution and seasonality of influenza viruses. Based on the characteristics of the morbidity of influenza in Ningbo, an ARIMA model for prediction was established.

## 2. Materials and Methods

### 2.1. Case Definition and Study Population

The study population included children (<15 years) and adults (≥15 years) who sought medical attention for ILI as an outpatient at three sentinel hospitals between January 2006 and December 2014. ILI was defined as a sudden onset of fever (≥38 °C) and cough or sore throat <5 days in duration for all ages [10].

### 2.2. Specimen and Data Collection

Nasopharyngeal swabs were collected, placed in 5 mL viral transport medium, stored at 4 °C, and transferred to the laboratory at the Ningbo Centers for Disease Control within 24 h. The original swabs were then preserved at −70 °C. Basic demographic and clinical information was also collected.

### 2.3. Laboratory Testing

Influenza viruses were isolated by inoculating specimens onto specific pathogen-free eggs or monolayers of Madin-Darby Canine Kidney cells in 2 mL of Dulbecco’s minimum essential medium (Sigma-Aldrich, St. Louis, MO, USA), supplemented with 3 μg/mL trypsin (Sigma-Aldrich). Influenza viruses were identified using a hemagglutination inhibition assay to determine the type and subtype of influenza A isolates using specific antisera, as recommended by the World Health Organization [11].

### 2.4. Ethics Statement

This study was determined to be a routine public health surveillance activity. Therefore, a formal ethical review was not required. The surveillance data and specimens were collected as part of the surveillance, and verbal informed consent was obtained from each subject. The patient identities have not been disclosed at any stage.

### 2.5. ARIMA Model

The ARIMA model was based on the monthly percentage of ILI (ILI%) during 2006–2014 in Ningbo, China. As a rule, the standard statistical methodology to establish an ARIMA model includes three steps: identification, parameter estimation, and diagnostic checking.

The identification stage involves the determination of the differencing requirement of making the time series stationary and the identification of the temporal structure of the model. Stationarity is a precondition for building an ARIMA model to transform the non-stationary time series into a stationary time series using differencing processes. D is the order of regular difference. D is the order of seasonal difference. An augmented Dickey-Fuller (ADF) test can determine whether the time series after differencing was stationary. Based on the graphs for the autocorrelation function (ACF) and partial autocorrelation function (PACF), we can identify the possible values of *p* (regular auto regressive), *q* (moving average), *P* (seasonal autoregressive), and *Q* (moving average). Generally, more than one tentative model is chosen in this step.

Parameters in the ARIMA model are estimated with the unconditional least squares method after the identification step [12]. The significant parameters are kept, and the others are excluded.

Finally, the adequacy of the established model for the series is verified using the Box-Jenkins *Q* test [13] to check whether the residuals are equivalent to white noise. Generally speaking, if the *p* value of the *Q*-statistic is not >0.8, the tentative model is inadequate. Then, the best ARIMA model is selected from the possible models using the Bayesian Information Criterion (BIC), where the fitted model is the one with the lowest BIC value. The fitted ARIMA model was used for short-term forecasting of ILI% for 2015 in this study.

### 2.6. Statistical Analysis

SPSS statistical software (SPSS Inc., Chicago, IL, USA) was used for data analysis and to create the ARIMA model and make predictions (Supplementary Materials). A *p-*value < 0.05 was considered statistically significant.

The ILI cases were divided into four age groups. To compare epidemiological factors, the epidemiological and laboratory data were analyzed using Pearson’s chi-squared tests. Correlation analysis was used to determine the rate of influenza virus isolation associated with the trend in ILI variation in the outpatients.

## 3. Results

### 3.1. Influenza Surveillance from 2006 to 2014

During 2006–2014, 129,528 ILI (3.15%) cases from 4,130,530 outpatient patients at the three sentinel hospitals were enrolled, ranging in age from 0 to 90 years. A high prevalence of ILI was present in all age groups (Table 1). The prevalence of ILI was the highest (80.99%) in patients aged <15 years and primarily affected patients aged 0–4 years. The lowest percentage of ILI (2.32%) was present in the group aged ≥60 years. The ILI prevalence was significantly different between the age groups (*χ*^2^ = 76.72, *p* < 0.05).

**Figure 1:**
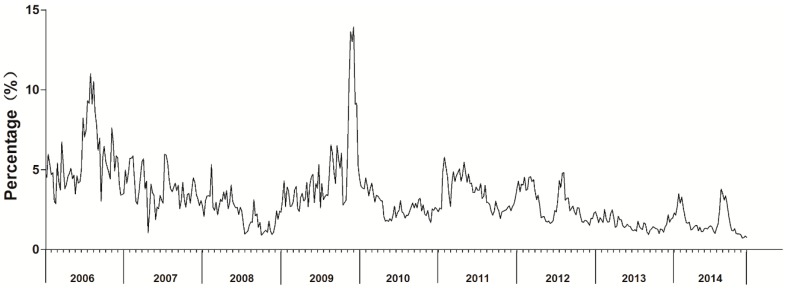
Monthly influenza-like illness rates in Ningbo, China, 2006–2014.

ILI% had roughly seasonal fluctuations and a slightly decreasing trend (Figure 1). In 2009, ILI% had two peaks of influenza activity, one in winter-spring and the other in summer. During the study period, the highest peaks and the lowest ILI% occurred in 2009 and 2014, respectively.

### 3.2. Viral Etiology of Patients with Influenza-Like Illness

Of the 13,294 specimens collected during the 9 years, 1148 (8.64%) were positive for influenza viruses. Of these, 173 (15.07%) were positive for seasonal A/H1N1 subtype, 416 (36.24%) for seasonal A/H3N2 subtype, 362 (31.53%) for pandemic A/H1N1 subtype, and 197 (17.16%) for influenza B virus (Table 2). There was no significant difference in the influenza virus positivity by year (*χ*^2^ = 3.66, *p* = 0.886).

**Figure 2:**
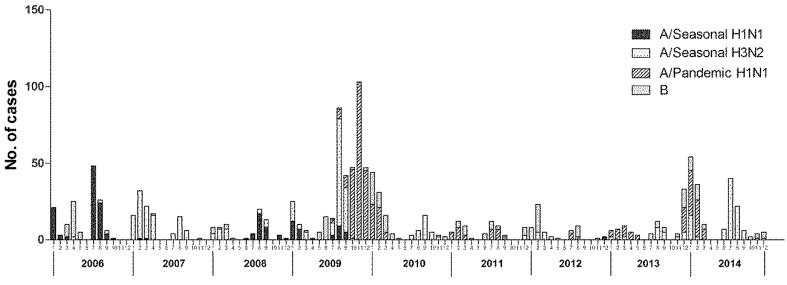
Time distribution of influenza subtypes in Ningbo, China, 2006–2014.

**Figure 3:**
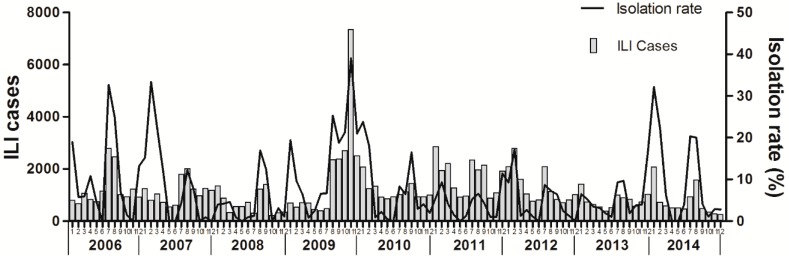
Number of patients with influenza-like illness and positivity rate of influenza viruses isolated by month in Ningbo, China, January 2006 to December 2014.

The dominant circulating virus in Ningbo continually changed. Seasonal A/H1N1 was the major circulating strain in 2006 and 2008. Seasonal A/H3N2 was the dominant circulating virus in 2007, while pandemic A/H1N1 was the predominant strain in 2009 and 2010. During 2006–2014, the predominant influenza virus subtypes that caused the two obvious peaks were seasonal A/H1N1 and A/H3N2 for 2006–2007 and pandemic A/H1N1 in the winter of 2009 (Figure 2). The influenza virus isolation rate was strongly correlated with the rate of ILI during the overall study period (*r* = 0.20, *p* < 0.05), and the changes in the trends were similar (Figure 3).

### 3.3. Time Series Analysis of Monitoring Data

Although the ARIMA model requires data to be stationary, the time series was not stationary (Figure 1; ADF test, *p* > 0.05). Three steps were used to obtain a stationary time series. First, the first-order non-seasonal difference (d = 1) was calculated. As a result, the ACF and PACF graphs indicated a high seasonal behavior with a circle of 12 (s = 12). Second, to remove the monthly seasonality, the first-order seasonal difference (d = 1) with a circle of 12 was calculated. Finally, the result of the ADF test was statistically significant (*p* < 0.05), confirming that the transformed time series was stationary.

**Figure 4:**
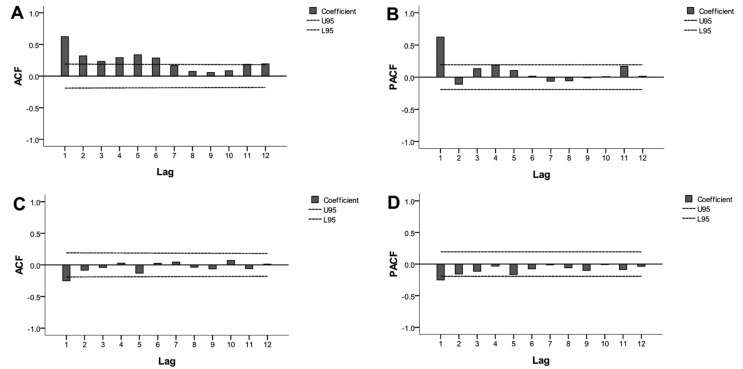
Autocorrelation function (ACF) and partial autocorrelation function (PACF) plotted against time lags for the original series (**A**,**B**, respectively) and after one order of regular differencing and one order of seasonal differencing (**C**,**D**, respectively). Dotted lines indicate the 95% confidence intervals (CIs). Most of the correlations fall around zero within their 95% CIs (U95: upper limit of 95% CI; L95: lower limit of 95% CI) except at the first lag, which indicates the series would achieve stationarity.

Further statistical analyses were performed with the stationary series. Four models were conducted as shown in Figure 4: ARIMA (1, 1, 0) (1, 1, 0)_12_, ARIMA (1, 1, 1) (1, 1, 0)_12_, ARIMA (0, 1, 0) (1, 1, 0)_12_, and ARIMA (0, 1, 1) (1, 1, 0)_12_. Based on parameter estimation and the goodness of fit test statistics (Table 3 and Table 4), we confirmed that the best model was ARIMA (1, 1, 1) (1, 1, 0)_12_. The Ljung-Box statistical test did not reject the null hypothesis of independence in the residuals’ time series (*Q* = 15.21, *P* = 0.44). Thus, the residuals’ error was considered to be a white noise sequence, and the selected model was confirmed to be appropriate.

**Figure 5:**
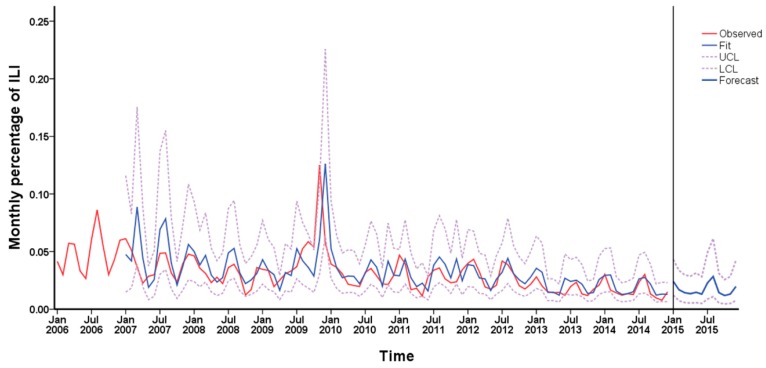
Time series profile for the prediction of influenza by the autoregressive integrated moving average (ARIMA) (1, 1, 1) (1, 1, 0)_12_ model.

Finally, the ARIMA model (1, 1, 1) (1, 1, 0)_12_ was used to forecast ILI% from January to December 2015. The fitting and forecasting results are shown in Figure 5. Compared with the actual values in 2015, the results of prediction were fitted well, indicating that the ARIMA model was effective.

## 4. Discussion

In this study, we present the first standardized surveillance data for influenza activity in Ningbo, China; the month with the peak influenza isolation rate was almost identical to that with the highest ILI%. Because ILI is a clinical definition designed to detect potential influenza cases, influenza viruses are most likely to be identified when ILI is used to define cases, and ILI is strongly correlated with the influenza virus isolation rate [14]. Hence, ILI is widely used as a sensitive indicator to reflect the situation of an influenza pandemic [15]. In our surveillance system, sample collection and laboratory testing for influenza surveillance in Ningbo, China were prompt and efficient.

The results of 9 years of longitudinal surveillance showed that influenza circulates each year in Ningbo with two clear activity peaks in November to February and July to September, corresponding with the epidemic regularity of influenza in southern China [16]. Consequently, the influenza virus vaccine should be considered for an annual immunization plan in specific populations before the period of virus activity in China.

We found that the periods of influenza activity in Ningbo overlapped with periods of increased rainfall or colder temperatures, despite the apparent lack of any significant correlation between the number of ILI cases and rainfall or any other environmental factors in this study, which was in line with observations in other subtropic regions [17]. The relatively high indoor humidity during the rainy season and prolonged survival of the influenza virus in aerosols during the winter months might contribute to the seasonal spread. However, these results differ from those in the tropical regions of Africa, where influenza activity has been reported to peak mostly in the rainy season [18]. This might contribute to the difference of latitudes [19], suggesting more studies should be carried out in the multicenter to explore influenza influence factors.

The ILI surveillance data also indicated that the trend in ILI% was similar every year during 2006–2014, with relatively stable fluctuations in peak amplitude. The obvious peaks in 2006 and 2009 were associated with a large epidemic or an outbreak of influenza, similar to the large-scale epidemic peak observed with the Victoria-like virus of influenza B that caused a flu outbreak at 12 schools in Ningbo from March to April 2006 [20]. The autumn peak in 2009 was largely influenced by the novel influenza A pH1N1 (pH1N1) activity, indicating the emergence of novel influenza viruses with pandemic potential [21], similar to the increased influenza activity during the pandemic period in Beijing, China [22]. After the pandemic period in 2009, the influenza epidemic intensity decreased in the following year because the population had acquired some level of protective antibodies after the pH1N1 infection.

All of the influenza A and B virus strains circulated during the study period, with the epidemic strain mutating over time. The seasonal A/H1N1 virus, which had become a part of the alternating seasonal influenza epidemics with other strains, was replaced by the pandemic A/H1N1 virus since its emergence in 2009. Of the influenza A subtypes, seasonal A/H3N2 (sH3N2, emerged in 1968) was the most prevalent prior to the appearance of pandemic A/H1N1 and circulated during the summer, similar to findings in Pakistan [23]. However, the number of cases with influenza B closely followed, in second place, the number of cases with pandemic A/H1N1. Therefore, the effect of a year-round presence of influenza B viruses on the health care system should be estimated concurrent with an analysis of the influenza A-associated morbidity and mortality, because we observed that both influenza A and B viruses co-circulated during the surveillance period. Also, B subtypes were often accompanied by prevalent influenza A infections and vice versa.

All age groups were susceptible to influenza; however, nearly half of the ILI cases were children aged <4 years old. Similarly, a previous risk factor analysis indicated that young age was a strong risk factor for ILI [24], and numerous studies have reported that most seasonal influenza viruses affect infants. Infants have weaker immune systems, and the symptoms of respiratory disease are similar to ILI in infants, which resulted in a higher number of ILI cases. Also, the infection or reinfection rate in children might have been higher than that in adults, resulting in more doctor visits for children than adults [14]. The number of ILI cases in the 5–14-year-old age group was also high; these represent school-aged children who are likely to have more opportunities to be infected because of close contact with peers and less consciousness regarding the need for self-protection. These findings contribute to the growing literature documenting that school-aged children experience high rates of severe respiratory infections [25,26,27,28]. In contrast to previous reports [29], the prevalence of ILI in patients aged ≥60 years was much lower than that in the other age groups. It is possible that older men were less prone to seek care for ILI. Accordingly, these findings could provide clues for health education and vaccination strategies for the target population.

Many natural and social environment factors affect the incidence of influenza, which leads to difficulties when forecasting using regression forecasting methods. However, the main advantage of a time series analysis for predicting the incidence of influenza is that it did not need to consider the effects of various factors. That is to say, the ARIMA model could perform the prediction only by the changing nature of the disease itself. The incidence of influenza was closely related with climate and the surveillance data from Ningbo. The significant seasonal changes were fitted using the ARIMA models, which were demonstrated as feasible for influenza prediction in this study.

The ARIMA (1, 1, 1) (1, 1, 0)_12_ model developed in this study attempts to provide a simple tool to predict the expected future number of ILI cases per month based on the observed ILI cases over the years. The actual data agreed with the predicted data from the ARIMA model, which can provide good results for the forecasting of ILI; therefore, the use of the ARIMA model is feasible for forecasting the incidence of influenza. However, the ARIMA model is generally used for short-term forecasts because the relative bias of prediction increases with time, resulting in poor long-term prediction. This might be explained by antigenic drift of the influenza virus and many unknown factors that affect the fluctuations in outbreaks such as improved detection methods and a higher frequency of vaccination. Because it is difficult to predict the long-term number of ILI cases, decisions for public health and the system of disease prevention should consider the comprehensive influence from other factors. However, short- and mid-term predictions of the influenza virus were possible owing to the procession for a new prevalent virus strain. This is encouraging regarding the ability to mimic the random procession of influenza virus antigen drift and consideration of other factors affecting the prevalence.

## 5. Limitations

Some limitations also should be addressed in our study. (1) The rainfall and other environmental factors were not considered in this study, which should be accounted for in further studies; (2) Other models such as integer-valued autoregressive (INAR) or similar models based on Poisson or negative binomials might be more effective in predicting influenza incidence, which were not mentioned in this paper.

## 6. Conclusions

In conclusion, our sustainable surveillance system was able to indicate the epidemiology and circulation of influenza viruses in Ningbo, China. Continuing influenza surveillance could constitute part of the epidemiological data required for preventive measures such as vaccination campaigns for high-risk groups in the Ningbo population. The ARIMA (1, 1, 1) (1, 1, 0)_12_ model was the best fit of the statistical models for predicting ILI cases. This information will also be useful for administrators in effectively implementing preventive and control measures for the influenza virus.

## Figures and Tables

**Table 1:** Distribution of the influenza-like illness cases by age group in Ningbo, China, 2006–2014.

Year	0~	5~	15~	25~	≥60	Total
	No. of ILI	No. of ILI	No. of ILI	No. of ILI	No. of ILI	
2006	7835 (52.76%) ^a^	3430 (23.10%)	957 (6.44%)	2388 (16.08%)	240 (1.62%)	14,850
2007	7181 (52.72%)	3559 (26.13%)	828 (6.08%)	1771 (13.00%)	282 (2.07%)	13,621
2008	4744 (53.57%)	1871 (21.13%)	854 (9.64%)	1175 (13.27%)	212 (2.39%)	8856
2009	9081 (40.39%)	7005 (31.16%)	2915 (12.97%)	3133 (13.94%)	347 (1.54%)	22,481
2010	6957 (49.51%)	2776 (19.75%)	1711 (12.18%)	2112 (15.03%)	497 (3.54%)	14,053
2011	9991 (48.28%)	4321 (20.88%)	2960 (14.30%)	3041 (14.70%)	380 (1.84%)	20,693
2012	8027 (47.69%)	2499 (14.85%)	2870 (17.05%)	3078 (18.29%)	359 (2.13%)	16,833
2013	3708 (40.52%)	1102 (12.04%)	1890 (20.66%)	2194 (23.98%)	256 (2.80%)	9150
2014	2989 (33.25%)	940 (10.46%)	1911 (21.26%)	2713 (30.18%)	437 (4.86%)	8990
Total	60,513 (46.72%)	27,503 (21.23%)	16,896 (13.04%)	21,605 (16.68%)	3010 (2.32%)	129,528

ILI, influenza-like illness; ^a^ indicated the constituent ratio of ILI.

**Table 2:** Results of the detection of the influenza virus from patients with influenza-like illness in Ningbo, China, 2006–2014.

Year	No. of Samples	No. of Positive	Positive Rate (%)	Influenza A Virus			Influenza B Virus
				H1N1	H3N2	pdm H1N1	
2006	1290	145	11.24	103 (71.03%)	6 (4.14%)	0 (0%)	36 (24.83%)
2007	1223	113	9.24	2 (1.77%)	109 (96.46%)	0 (0%)	2 (1.77%)
2008	1230	56	4.56	31 (55.36%)	19 (33.93%)	0 (0%)	6 (10.71%)
2009	2359	401	17.00	37 (9.23%)	129 (32.17%)	209 (52.12%)	26 (6.48%)
2010	1821	128	7.03	0 (0%)	31 (24.22%)	49 (38.28%)	48 (37.50%)
2011	864	26	3.01	0 (0%)	3 (11.54%)	11 (42.31%)	12 (46.15%)
2012	866	24	2.77	0 (0%)	6 (25.00%)	0 (0%)	18 (75.00%)
2013	1658	69	4.16	0 (0%)	18 (26.09%)	33 (47.83%)	18 (26.09%)
2014	1983	186	9.38	0 (0%)	95 (51.08%)	60 (32.26%)	31 (16.67%)
Total	13,294	1148	8.64	173 (15.07%)	416 (36.24%)	362 (31.53%)	197 (17.16%)

**Table 3:** Parameter estimation for available autoregressive integrated moving average (ARIMA) models for the prediction of influenza.

Parameter	ARIMA (1, 1, 0) (1, 1, 0)_12_			ARIMA (1, 1, 1) (1, 1, 0)_12_			ARIMA (0, 1, 0) (1, 1, 0)_12_			ARIMA (0, 1, 1) (1, 1, 0)_12_		
	*SE*	*t*	*P*	*SE*	*t*	*P*	*SE*	*t*	*P*	*SE*	*t*	*P*
Constant	0.002	−0.191	0.849	0.000	−0.491	0.625	0.002	−0.155	0.877	0.002	−0.224	0.823
AR1	0.103	−1.386	0.169	0.104	5.984	0.000	-	-	-	-	-	-
MA1	-	-	-	1.835	0.544	0.588	-	-	-	0.098	3.347	0.001
SAR1	0.084	−6.590	0.000	0.086	−6.363	0.000	0.084	−6.520	0.000	0.084	−6.623	0.000

SE, standard error; AR, autoregressive parameter; MA, moving average parameter; SAR, seasonal autoregressive parameter.

**Table 4:** Goodness of fit statistics for plausible autoregressive integrated moving average (ARIMA) models for the prediction of influenza.

Statistic	RMSE	MAE	MAPE	BIC
ARIMA (1, 1, 0) (1, 1, 0)_12_	0.016	0.009	31.663	−8.181
ARIMA (1, 1, 1) (1, 1, 0)_12_	0.014	0.009	28.785	−8.311
ARIMA (0, 1, 0) (1, 1, 0)_12_	0.016	0.009	31.701	−8.197
ARIMA (0, 1, 1) (1, 1, 0)_12_	0.015	0.009	31.984	−8.230

RMSE, root mean square error; MAE, mean absolute error; MAPE, mean absolute percentage error; BIC, bayesian information criterion.

## Supplementary Materials

The following are available online at www.mdpi.com/1660-4601/14/6/559/s1.
