# Supplementary material for: Epidemiological Features and Forecast Model Analysis for the Morbidity of Influenza in Ningbo, China, 2006–2014
Source: Int J Environ Res Public Health. 2017 May 25;14(6):559. doi: 10.3390/ijerph14060559 (PMC5486245; doi:10.3390/ijerph14060559)
Supplement: Supplementary file 1 [file ijerph-14-00559-s001.pdf]

International Journal of
Environmental Research and Public Health

MDPI

Supplementary Materials: Epidemiological Features and Forecast Model Analysis for the Morbidity of Influenza in Ningbo, China, 2006–2014

Chunli Wang [1,†], Yongdong Li [2,†],Wei Feng [1], Kui Liu [3], Shu Zhang [2], Fengjiao Hu [2], Suli Jiao [2], Xuying Lao [2], Hongxia Ni [2] and Guozhang Xu [2,*]

The process of creating an ARIMA model by SPSS statistical software was listed as follows:
(1) From the menu bar of SPSS, select the "Analyze" | "Time series" | "Create models" command, then open the dialog box of "time series modeling".
(2) Selection of variables and methods. Select the dependent variable that creates the ARIMA model from the source variable list. Then choose "ARIMA" in the method's drop-down list box.
(3) Set the settings accordingly, including Statistics setting, Chart setting, Output filter settings, Save settings and Option setting.

Specific procedures can refer the related reference below :

1. Zhang W.T. *The Course of Statistical Analysis with SPSS*; Hope Electronic Press: Beijing, China, 2002, pp. 250–289. (In Chinese)
2. Chen, S.K. *SPSS Statistical Analysis from Entry to Mastery;* Tsinghua University Publishing House: Beijing, China, 2013, pp. 398-401. (In Chinese)

© 2016 by the authors; licensee MDPI, Basel, Switzerland. This article is an open access article distributed under the terms and conditions of the Creative Commons by Attribution (CC-BY) license (http://creativecommons.org/licenses/by/4.0/)
